# Quantitative analysis of facial asymmetry based on three-dimensional photography: a valuable indicator for asymmetrical temporomandibular joint affection in juvenile idiopathic arthritis patients?

**DOI:** 10.1186/s12969-020-0401-y

**Published:** 2020-01-31

**Authors:** Joëlle M. Bernini, Christian J. Kellenberger, Martina Eichenberger, Theodore Eliades, Spyridon N. Papageorgiou, Raphael Patcas

**Affiliations:** 1 Clinic of Orthodontics and Paediatric Dentistry, Center of Dental Medicine, University of Zürich, Plattenstrasse 11, 8032 Zurich, Switzerland; 2 Department of Diagnostic Imaging, University Children’s Hospital Zürich, Zurich, Switzerland

**Keywords:** Juvenile idiopathic arthritis, Temporomandibular joint, Facial asymmetry, Stereophotography, Three-dimensional photography, Morphometric analysis

## Abstract

**Background:**

Juvenile idiopathic arthritis (JIA) can cause osseous deformity in the temporomandibular joint (TMJ) and may impair mandibular growth. This study aimed to evaluate whether facial asymmetry determined clinically or by morphometric analysis of three-dimensional (3D) photographs in JIA patients is associated with an asymmetric affection of theTMJ.

**Methods:**

Of 76 consecutive JIA patients with a mean age of 11.7 years (range: 6.3–17.9), facial asymmetry was evaluated clinically (chin asymmetry, gonion asymmetry), and stereophotogrammetrically with 3D photographs. The facial surfaces were demarcated, then mirrored, superimposed using semi-automated landmarks, and quantitatively assessed (chin asymmetry, Hausdorff distances). Clinical and digital measurements were related to the diagnosis of right and left TMJ involvement derived from magnetic resonance images (MRI).

**Results:**

Twenty-seven (34%) patients had an asymmetrical osseous deformity of the TMJ. By clinical evaluation, chin asymmetry was related to asymmetrical osseous destruction (*p* = 0.02), but gonion asymmetry was not (*p* = 0.14). In regard to 3D-photograph based morphometric measurements, chin asymmetry was also related to asymmetrical osseous destruction (*p* = 0.01), but neither the mean (*p* = 0.06) nor the maximal Hausdorff distance (*p* = 0.67). Despite the attested significance, none of the chin asymmetry evaluation methods appeared to hold sufficient predictive value (positive predictive values ≤54%; coefficient of determination ≤7%).

**Conclusions:**

For the assessment of facial asymmetry in JIA patients, morphometric measurements originating from 3D-photographs seem to deliver results comparable to the clinical assessment methods. The asymmetry of the face, especially around the chin, appears to be related to asymmetrical TMJ destruction, but none of the investigated measurement methods of the face were able to reliably predict the TMJ affection. Thus, facial asymmetry assessments, both qualitatively in a clinical setting and quantitatively based on 3D-photographs, have limited diagnostic value for TMJ involvement in JIA patients.

## Background

Juvenile idiopathic arthritis (JIA) is a term that encompasses all forms of autoimmune, non-infective inflammatory joint diseases of unknown aetiology with an onset before the age of 16 years [1]. It is the most common rheumatoid disorder in childhood [2], with an estimated involvement of the temporomandibular joint (TMJ) in approximately 40–96% of the children [3].

The inflammatory activity in the TMJ is seen as the source of two distinct morphopathologies: Osteochondral lesions within the joint itself, and - since the TMJ is also an important centre of growth during childhood - craniofacial growth disturbances [4]. Patients whose TMJs are not affected to the same degree on both sides are expected to develop dentofacial asymmetry [5, 6].

While several diagnostic approaches, such as ultrasound examination or the evaluation of panoramic films and cephalograms, have been suggested and ultimately questioned [7] in the past, contrast-enhanced magnetic resonance imaging (MRI) remains undisputedly the ideal diagnostic tool for the assessment of inflammatory activity and osteochondral degradation of the TMJ [8–10].

Previous reports indicate that certain facial morphological features, such as an antegonial notching or chin asymmetry, could be interpreted as signs of impaired growth representing a structural damage in the TMJ [11, 12]. As arthritis of the TMJ is frequently asymptomatic, a dependable facial examination could serve an important purpose to disclose possible craniofacial growth deficiency and to establish a timely interventional strategy [13]. With the introduction of three-dimensional (3D) photography as documentation method for facial morphology [14], it is assumed that the diagnostic value of facial assessment to detect TMJ involvement can be increased. Indeed, the advocated advantages of an evaluation of the face by means of stereophotogrammetry include not only an improved reproducibility, high spatial resolution, and no ionizing radiation, but also the possibility to conduct quantitative analyses from morphometric measurements.

The clinical relevance of linking facial diagnostics to TMJ deformation is evident, as it would facilitate a timely intervention for craniofacial growth deficiency treatment. The purpose of this study was therefore to investigate in JIA patients whether an association between facial assessment – either clinical or stereophotogrammetrical – and osseous deformity in the TMJ – as attested on MRI – could be detected. More specifically, the aims were (1) to disclose any potential associations between facial asymmetry and an asymmetrical osseous destruction of the TMJ, (2) to evaluate the predictive value of different facial examinations, and (3) to disclose possible relationships between 3D-photography based facial measurements and the administered drugs.

## Methods

### Patients

This is a retrospective study of patients diagnosed for JIA (according to the International League of Associations for Rheumatology criteria [15]) seen jointly at the Clinic for Orthodontics and Paediatric Dentistry of the local university and the University Children’s hospital during the years of 2017 and 2018. Inclusion criteria were complete clinical records comprising a MRI of the TMJ, a 3D-photography of the face, a clinical assessment, and the medical history of the patients. The MRI had to be performed at a maximal interval of 3 months to the 3D-photography and the clinical assessment (which were both always taken on the same day). Children with no consent for retrospective data analysis were not considered.

Patient histories were screened and drugs administered for JIA treatment were recorded as none, “systemic” (i.e., systemic immunosuppressive therapy), or “local” (i.e., local corticosteroid TMJ injection).

### MRI evaluation of the TMJ

In adherence to the institutional protocol [16] and in accordance with the evidence-base [8], MRI evaluation of the TMJ was conducted on contrast-enhanced sequences performed at 1.5 Tesla (Discovery MR450, GE Medical Systems, Milwaukee, USA) with a TMJ surface coil in closed mouth position, performed at the local University Children’s hospital. A systematic evaluation of the TMJ affection was performed by an experienced board certified paediatric radiologist (three levels: TMJ not affected, inflammatory activity without osseous destruction, inflammatory activity with osseous destruction), for each joint independently, based on the criteria of established progressive scoring systems both for the *level of inflammation* and the *degree of osteochondral deformation* [17–19]. *Inflammatory activity* was assessed on fat-saturated T2-weighted images based on the presence and degree of joint effusion, synovial thickening, and bone marrow oedema, in addition to contrast-enhanced images for the presence and extension of joint enhancement. *Osseous deformity* was established on gradient echo images, identifying the shape and integrity of the temporal bone (articular eminence and glenoid fossa) and mandibular condyle.

### Clinical and digital assessment of facial asymmetry

Clinical assessment of facial asymmetry was performed by an experienced board certified orthodontist. Facial asymmetry was established in cases of palpable differences at the antegonial notching (“Clinical Gonion Asymmetry”: present or absent) and of evident chin deviation (“Clinical Chin Asymmetry”: present or absent) [11].

Digital assessment of facial asymmetry was based on the 3D-photographs (Vectra 3 M, Canfield Scientific, New Jersey, USA) of the patients, produced as part of the annual check-up. The stereophotographs were taken while the patients were seated with the face positioned according to the Frankfurt horizontal plane. The patients were instructed to maintain a neutral facial expression in maximal intercuspidation. The 3D photographic images were processed with an open source software (3D Slicer, Release 4.4.0) [20] and several extension modules (in order of application: EasyClip, CMFreg, Model To Model Distance, Mesh Statistics Extension, ShapePopulationViewer) [21]. The file format of the stereophotographs was transformed from object (obj) files to visualization toolkit (vtk) files and edited by removing neck, hair, and the forehead, 1 cm above the eyebrows.

**Fig. 1:**
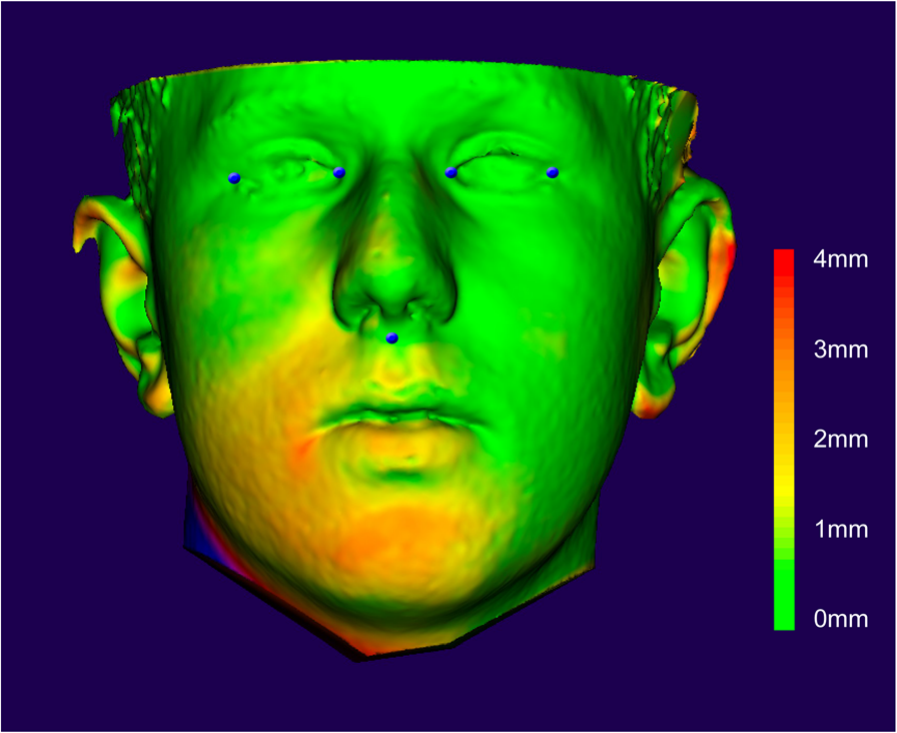
Representative visualization of mandibular asymmetry based on the superimposition of the mirrored surface of the face. Registration was performed with 5 landmarks of the midface (blue dots), and asymmetry was calculated for the chin and the entire lower face, separately

In order to determine the asymmetry of the lower face, the faces were mirrored and superimposed for a best-match registration of the midface, using five semi-automated landmarks (i.e., subnasal, bilateral inner and outer angle of the eye). Following the landmarks registration, the Hausdorff distance (HAD) between the original and the mirror picture was measured for the cropped lower face as region of interest, and mean HAD as well as maximum HAD were recorded for every patient separately. Asymmetry of the chin area was visualized using a colour coding for the distance of the mirrored faced to the original face (see Fig. 1).

All examiners (MRI evaluation, clinical assessment and analysis of the 3D-photography) were blinded to the results of the other examination methods. Twenty randomly selected cases were re-measured, three months apart, to establish repeatability.

### Statistical methods

Descriptive statistics were calculated for all variables, the distribution of the continuous variables (HAD mean, HAD max., digital chin asymmetry) was verified with a Shapiro-Wilk test, and intraclass correlation coefficients (ICC) for absolute agreement were calculated to confirm repeatability of the measurements. Contingency tables were computed to reveal possible associations between the MRI diagnosis and the asymmetry results, together with Pearson’s chi-squared tests and positive predictive values for categorical variables. A ROC analsys was performed to detect the value of digital chin asymmetry with best discriminative ability to discern patients with TMJ involvement. Possible relationships between the continuous results of the digital assessments and the MRI diagnosis were investigated with a Mann-Whitney U-test, and the predictive value for each continuous variable was established by calculating the Cox & Snell coefficient of determination based on binary logistic regression models. Contingency tables and Mann-Whitney U-tests were also applied to disclose potential associations between the treatment modality and the results of the digital assessment. The significance level for all statistical tests was set to a *p*-value (p) < 0.05. All analyses were run in SPSS (version 24.0; IBM Corp., Armonk, New York, USA).

## Results

Seventy-six patients (50 females, 65.8%) were identified and analysed. Patient data, results of clinical examinations and administred JIA-related medication since diagnosis were retrieved from patient files. JIA-Subtype and disease duration are summarised in Table 1. The patients’ age at the clinical assessment / 3D-photography ranged from 6.3 to 17.9 years with a mean age of 11.7 years.

**Table 1:** Demographic characteristics of the 76 consecutive patients analysed in this study

JIA-Subtype	N	%
Oligoarticular	42	55.3
Oligoarticular extended	6	7.9
Polyarticular RF negative	16	21.0
Enthesitis-related arthritis	3	3.9
Psoriasis arthritis	4	5.3
Systemic	1	1.3
Not classified	4	5.3
Age	Median (y)	IQR (y)
Age at diagnosis	4.5	2.7–7.0
Age at clinical assessment and 3D-photography	11.7	9.6–14.0
Disease duration	5.8	3.4–9.4

*N* number of patients; *IQR* interquartile range

Of all 76 patients, 60 (75.9%) were administered medicinal therapy for JIA treatment: 31 (40.8%) received systemic immunosuppressive drugs, and 29 (38.2%) were subject to local corticosteroid TMJ injection.

The repeatability of the digital measurements was high, with slightly better agreement for chin asymmetry (ICC: 1.0) than for the Hausdorff distances, HAD max (ICC: 0.95) or HAD mean (ICC: 0.81). The reliability of the applied MRI scoring system for inflammatory activity and osteochondral deformity is uncontested and has been documented elsewhere [19].

**Fig. 2:**
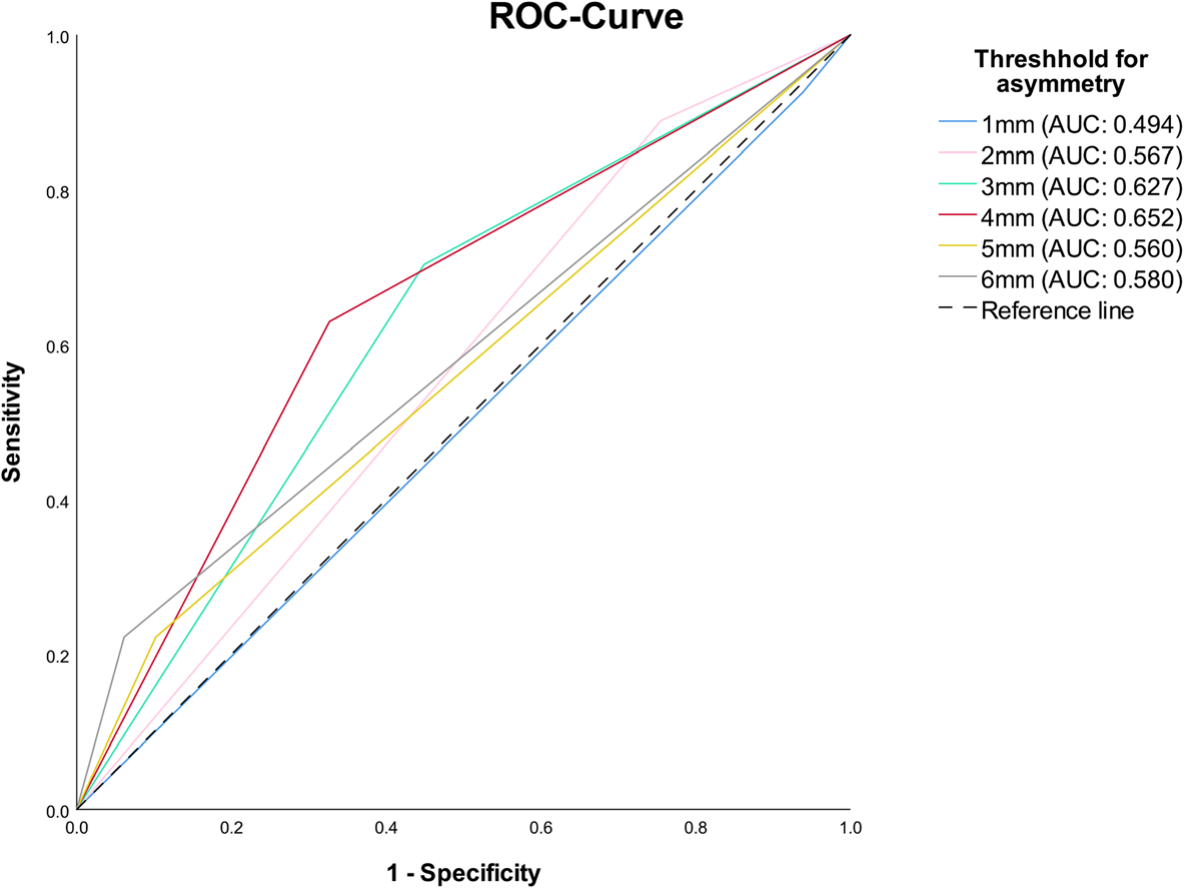
ROC-curves illustrating the discriminatory power of the different digital chin asymmetry levels (1 mm – 6 mm) to predict an asymmetrical osseous destruction diagnosed on the MRI. AUC: Area under the curve

The descriptive results of the MRI observations are shown in Table 2. Approximatively 50% of the assessed joints showed inflammatory activity without osseous affections, and an osseous deformity was diagnosed in further 25% (left) to 29% (right) of the cases. The outcomes of the asymmetry assessments are summarized in Table 3. Chin asymmetry was clinically observed in 32% of the cases, and the digital assessment resulted in a mean asymmetry of 3 mm (range: 0 mm – 6 mm). According to the ROC analysis (Fig. 2), 4 mm proved to be the amount of digital chin asymmetry with the highest discriminative power (Area under Curve: 0.652). Thus, for further testing, digital chin asymmetry was binned in < 4 mm and ≥ 4 mm. Of all 76 patients, 33 (43.4%) had a digital chin asymmetry of at least 4 mm. None of the continuous variables (digital chin asymmetry and HAD) followed normal distribution (*p* < 0.001).

**Table 2:** Descriptive statistics of the analysed sample

Factor	Na	N (%)
MRI		
Left TMJ not affected	76	19 (25.0%)
Left TMJ inflammatory activity without osseous destruction	76	37 (48.7%)
Left TMJ inflammatory activity with osseous destruction	76	20 (26.3%)
Right TMJ not affected	76	15 (19.7%)
Right TMJ inflammatory activity without osseous destruction	76	38 (50.0%)
Right TMJ inflammatory activity with osseous destruction	76	23 (30.3%)
Left and right TMJ not affected	76	12 (15.8%)
TMJ asymmetrical inflammatory activity	76	31 (39.2%)
TMJ asymmetrical osseous destruction	76	27 (34.2%)

*Na* eligible patients

**Table 3:** Assessment of Asymmetry: Clinical examination and measurements based on 3D-photography (“Digital examination”)

Factor	Na	N (%)		
Clinical Examination				
Clinical Chin Asymmetry	76	24 (31.6%)		
Clinical Gonion Asymmetry	76	45 (59.2%)		
Factor	Na	Mean (SD) mm	Median (IQR) mm	Range mm
Digital Examination				
Digital Chin Asymmetry	76	3.0 (1.7)	3.0 (2.0)	0–6.0
HAD mean	76	1.7 (0.6)	1.6 (0.8)	0.7–3.7
HAD maximum	76	16.6 (8.6)	14.1 (7.9)	6.1–57.7

*HAD* Hausdorff Distance; *IQR* interquartile range; *Na* eligible patients; *SD* standard deviation

The relationship between the various facial asymmetry assessments and an asymmetrical osseous destruction in the TMJ (as observed in the MRI) are investigated in Table 4 for categorical results and in Table 5 for the reported distances. The correlations between asymmetrical osseous destruction and facial asymmetry were statistically significant for clinical chin asyemmetry (*p* < 0.02) and digital chin asymmetry (both as categorical and continuous variable: *p* < 0.01). Nevertheless, none of the evaluated facial assessment seem to hold sufficient diagnostic value: the predictive value (≤54%) and the coefficient of determination (< 8%) of all facial assessments (clinical or digital) remained disappointingly low.

**Table 4:** Comparison of the facial asymmetry results – presented as binary outcome - among patients diagnosed with or without asymmetrical destruction between the right and left TMJ in the MRI

MRI diagnosis of TMJ		Clinical: Gonion Asymmetry		Clinical: Chin Asymmetry		Digital: Chin Asymmetry (≥ 4 mm)	
Asymmetrical osseous destruction	Na	n (%)	P*	n (%)	P*	n (%)	P*
No	49	26 (53%)	0.14	11 (22%)	0.02	16 (31%)	0.01
Yes	27	19 (70%)		13 (48%)		17 (63%)	
Positive predictive value			42%		54%		52%

*MRI* magnetic resonance imaging; *n* patients in this category; *Na* patients eligible; *TMJ* temporomandibular joint

*P value originating from Pearson’s chi-squared test

**Table 5:** Comparison of the facial asymmetry results – presented as continuous outcome - among patients diagnosed with or without asymmetrical destruction between the right and left TMJ in the MRI. The coefficient of determination (Cox & Snell R^2^) is derived from a binary logistic regression analysis predicting the asymmetrical destruction from each continuous variable independently

MRI diagnosis of TMJ		Digital: Chin Asymmetry (continuous, mm)		Digital: Mean HAD (mm)		Digital: Max. HAD (mm)	
Asymmetrical osseous destruction	Na	Median (IQR)	P^+^	Median (IQR)	P^+^	Median (IQR)	P^+^
No	49	2.0 (3.0)	0.01	1.5 (0.6)	0.06	14.3 (10.4)	0.67
Yes	27	4.0 (2.0)		1.7 (0.9)		13.7 (5.3)	
Coeff. of determination			7.3%		4.1%		0.7%

*HAD* mean Hausdorff Absolute Distance; *IQR* interquartile range; *MRI* magnetic resonance imaging; *TMJ* temporomandibular joint

^*+*^*P value originating from Mann-Whitney U-test*

The associations between the various digital asymmetry assessments and the treatment options received are explored in Table 6, in which an association between chin asymmetry and a past administration of local corticosteroid injection is disclosed.

**Table 6:** Comparison of the 3D-photography based digital asymmetry results among patients with or without TMJ treatment

		Digital: Chin Asymmetry (continuous, mm)			Digital: Chin Asymmetry (≥ 4 mm)		
**Treatment**		**n**	**Median (IQR)**	**P**^**+**^	**n**	**n (%)**	P*
Any	No	16	2.0 (2.0)	0.75	16	6 (38%)	0.59
	Yes	60	3.0 (2.0)		60	27 (45%)	
Local (with or without systemic drugs)	No	47	2.0 (3.0)	0.03	47	17 (36%)	0.07
	Yes	29	4.0 (2.0)		29	16 (55%)	

^*+*^* P value originating from Mann-Whitney U-test*

* P value originating from Pearson’s chi-squared test

## Discussion

This is the first study to assess three-dimensional (3D) facial morphology in patients with JIA in direct comparison to TMJ involvement ascertained by MRI diagnostics. It is seemingly also the first attempt to conduct a direct comparison between clinical assessment and stereophotogrammetric measurements for facial asymmetry in JIA patients.

To detect inflammation activity and early joint damage is a primordial diagnostic goal with direct impact on the treatment strategy. The development of new combination treatment strategies and the introduction of biologic response modifying drugs have reformed the management of juvenile idiopathic arthritis (JIA) towards early aggressive interventions [22]. These advances have substantially amplified the prospect of achieving disease remission or, at least, minimize the levels of disease activity, but underscore the need for rapid diagnosis and initiation of treatment [23]. Yet timely diagnosis is routinely complicated owing to the fact that the course of JIA, characterized as monocyclic, polycyclic or chronic, remains decidedly variable.

The prospect of being able to use any clinical assessment of facial features as a rapid, non-expensive and non-invasive diagnostic tool to detect an ongoing process in the TMJ has prompted several studies [6, 11, 12, 24–28]. While certain clinical tests aim to disclose the inflammatory activity in the TMJ, the evaluation of face morphology and asymmetry targets to reveal structural damage in the TMJ and its association to impaired growth. The literature on the diagnostic value of clinical facial assessment to detect TMJ involvement in JIA patients indicates that statistically significant positive correlations between clinical findings and MRI diagnosis indeed exist [11, 26]. More specifically, previous investigators stated that antegonial notching and chin asymmetry could allegedly be interpreted as signs of impaired growth representing a structural damage in the TMJ [11, 12].

The present findings portray in agreement with these reports an evident significant relationship between osseous lesions in the TMJ and clinically detectable facial asymmetry, especially for the chin. The results clearly indicate that the chin region is more indicative than any assessment of the shape of the mandible, and suggest that the degree of asymmetry should be measured at the chin and neither at the gonion region nor through the stereophotogrammetrically acquired Hausdorff distance.

Yet despite the observed statistical significance, which clearly demonstrate a relationship between face morphology and structural damage in the TMJ, the suitability of the clinical assessment *as a test* was called into question by some of the above-mentioned authors themselves, either because the low sensitivity such a test would yield [26], or because the statistical significance was lost when other factors were added to the equation [11]. Our results corroborate the questionable usefulness of facial assessment as a diagnostic tool. Although it is apparent that chin asymmetry correlated significantly to an asymmetrical bone destruction, the predictive value of facial asymmetry remained disappointingly low.

This study’s main goal was to evaluate the benefits of facial assessment derived from stereophotogrammetric technology, grounded on the assumption that the diagnostic value could be increased through a quantitative analysis of morphometric measurements. The hypothesis that quantitative analyses can improve diagnostics could not be substantiated.

Limited reports exist on the relationship between 3D facial photographs of JIA patients and presumed uni- or bilateral TMJ involvement established on cephalograms [14] or panoramic films [29, 30]. As outlined in the introduction, scientific literature has produced ample and rich evidence that these two-dimensional radiographs fail to reflect TMJ lesions and their extent reliably [7]. This has been acknowledged by the authors of the previous reports who stressed the pivotal necessity of studies that would base the TMJ involvement on MRI diagnostics [29]. The use of MRI offers not only superior diagnostic accuracy, but allows to grade the osseous lesions reliably and as such permits to distinguish between the *degree* of destruction, an aspect that has been thitherto blatantly neglected for bilateral TMJ involvement. Disregarding the shortcomings of the earlier studies and applying the mandatory reservations when juxtaposing the results of different research projects, the mean 3.5 mm chin deviation for unilaterally affected TMJ reported by the earlier work [30] seems comparable to the 4 mm median deviation confirmed in our study. Yet in stark contrast to those who conclude that a significant relationship could serve as validation of a testing method, this present study provides a more comprehensive analysis. Calculating the coefficient of variance to reveal how well the different measurements of asymmetry can explain an asymmetrical TMJ destruction, it is unambiguously evident that the confirmed significant associations do not comprise enough predictive power.

In order to enable a direct comparison to the clinical evaluation, digital chin asymmetry was categorized into a binary variable. Defining a threshold value for the asymmetry assessment was necessary, since the subclinical asymmetry present in literally all individuals [31] had to be excluded. Pursuant to the descriptive values and the ROC analysis, 4 mm seemed to provide the best discriminating cutpoint. One should however be cognizant that dichotomizing continuous variables is intrinsically problematic, as it leads to loss of information and degradation of statistical power [32]. This became obvious in the results concerning the relationship between the assessment of the treatment options received and the various digital asymmetry assessments: the continuous variable of chin asymmetry was significantly associated to local corticosteroid treatment, an observation lost in the binary variable.

The discovered relationship between digital chin asymmetry and a past administration of local corticosteroid injection is open to interpretation. The observational nature of this study does obviously not allow to establish any causality, yet the following hypothesis may be submitted: According to an earlier study [16] corticosteroid injection may possibly cause a progression of TMJ osseous destruction. Thus, the corticosteroid injection could per se be the reason for the growth disturbance of the face. Alternatively, it cannot be ruled out that corticosteroid injections were given specifically in cases with more severe destruction. Either way, the current results seem to be in line with the assumption that intra-articular corticosteroid injections in children with JIA do apparently neither preserve normal growth, nor do they prevent TMJ deformity [16].

### Limitations

This investigation suffers from its retrospective nature, its single-centre setting, its cross-sectional approach and the adequate yet restricted amount of individuals. Moreover, it should be recalled that the included patients underwent MRI for TMJ diagnosis specifically because a TMJ involvement was suspected, and were therefore more likely to present clinical signs (asymmetry or others) than other JIA subjects. Lastly, its attempted goal to discern a relationship between face asymmetry and asymmetrically affected TMJ is modest, as any clinical findings of an alteration of face morphology showing a correlation with the MRI finding would only indicate that structural damage in the TMJ has already occurred. Nevertheless, this investigation is important, as it provides crucial information on the applicability of 3D-photograph based morphometric analyses of the face in JIA patients, offers a direct comparison to established clinical assessments, and delivers a cautionary interpretation of significant correlations between face measurements and TMJ lesions.

On a last note, questioning digital assessment as a diagnostic tool to predict a TMJ involvement in a cross-sectional setting does not disqualify stereophotogrammetric based analyses of intra-individual changes. Future research attempts should therefore focus on exploring longitudinal data with serial 3D records of JIA patients.

## Conclusions

This is the first study to compare a detailed facial assessment of JIA patients with TMJ involvement as presented on MRI. The findings suggest that a relationship between facial asymmetry and asymmetrical TMJ involvement exist, especially when facial asymmetry is quantified by means of 3D-photography based measurements. The results indicate, however, that all explored facial assessment methods have very limited power to predict an asymmetrical TMJ involvement and are therefore not suitable as diagnostic tools.

## Data Availability

The datasets are available from the corresponding author upon reasonable request.

## Abbrevations

3D: Three-dimensional
HAD: Hausdorff distance
ICC: Intraclass correlation coefficient
JIA: Juvenile idiopathic arthritis
MRI: Magnetic resonance images
TMJ: Temporomandibular joint

## References

[1] Prakken B, Albani S, Martini A (2011). Juvenile idiopathic arthritis. Lancet.

[2] Goldmuntz EA, White PH (2006). Juvenile idiopathic arthritis: a review for the pediatrician. Pediatr Rev.

[3] Stoll ML, Kau CH, Waite PD, Cron RQ (2018). Temporomandibular joint arthritis in juvenile idiopathic arthritis, now what?. Pediatr Rheumatol Online J.

[4] Arvidsson LZ, Fjeld MG, Smith HJ, Flato B, Ogaard B, Larheim TA (2010). Craniofacial growth disturbance is related to temporomandibular joint abnormality in patients with juvenile idiopathic arthritis, but normal facial profile was also found at the 27-year follow-up. Scand J Rheumatol.

[5] Sidiropoulou-Chatzigianni S, Papadopoulos MA, Kolokithas G (2001). Dentoskeletal morphology in children with juvenile idiopathic arthritis compared with healthy children. J Orthod.

[6] Stabrun AE, Larheim TA, Höyeraal HM, Rösler M (1988). Reduced mandibular dimensions and asymmetry in juvenile rheumatoid arthritis. Arthritis Rheum.

[7] El Assar de la Fuente S, Angenete O, Jellestad S, Tzaribachev N, Koos B, Rosendahl K (2016). Juvenile idiopathic arthritis and the temporomandibular joint: a comprehensive review. J Craniomaxillofac Surg.

[8] Kristensen KD, Stoustrup P, Kuseler A, Pedersen TK, Twilt M, Herlin T (2016). Clinical predictors of temporomandibular joint arthritis in juvenile idiopathic arthritis: a systematic literature review. Semin Arthritis Rheum.

[9] Kuseler A, Pedersen TK, Herlin T, Gelineck J (1998). Contrast enhanced magnetic resonance imaging as a method to diagnose early inflammatory changes in the temporomandibular joint in children with juvenile chronic arthritis. J Rheumatol.

[10] Weiss PF, Arabshahi B, Johnson A, Bilaniuk LT, Zarnow D, Cahill AM (2008). High prevalence of temporomandibular joint arthritis at disease onset in children with juvenile idiopathic arthritis, as detected by magnetic resonance imaging but not by ultrasound. Arthritis Rheum.

[11] Keller H, Muller LM, Markic G, Schraner T, Kellenberger CJ, Saurenmann RK (2015). Is early TMJ involvement in children with juvenile idiopathic arthritis clinically detectable? Clinical examination of the TMJ in comparison with contrast enhanced MRI in patients with juvenile idiopathic arthritis. Pediatr Rheumatol Online J.

[12] Kjellberg H, Fasth A, Kiliaridis S, Wenneberg B, Thilander B (1995). Craniofacial structure in children with juvenile chronic arthritis (JCA) compared with healthy children with ideal or postnormal occlusion. Am J Orthod Dentofac Orthop.

[13] Niibo P, Pruunsild C, Voog-Oras U, Nikopensius T, Jagomagi T, Saag M (2016). Contemporary management of TMJ involvement in JIA patients and its orofacial consequences. EPMA J.

[14] Hsieh Y-J, Darvann TA, Hermann NV, Larsen P, Liao Y-F, Bjoern-Joergensen J (2016). Facial morphology in children and adolescents with juvenile idiopathic arthritis and moderate to severe temporomandibular joint involvement. Am J Orthod Dentofac Orthop.

[15] Petty RE, Southwood TR, Manners P, Baum J, Glass DN, Goldenberg J (2004). International league of associations for rheumatology classification of juvenile idiopathic arthritis: second revision, Edmonton, 2001. J Rheumatol.

[16] Lochbuhler N, Saurenmann RK, Muller L, Kellenberger CJ (2015). Magnetic resonance imaging assessment of Temporomandibular joint involvement and mandibular growth following corticosteroid injection in juvenile idiopathic arthritis. J Rheumatol.

[17] Kellenberger CJ, Abramowicz S, Arvidsson LZ, Kirkhus E, Tzaribachev N, Larheim TA. Recommendations for a Standard Magnetic Resonance Imaging Protocol of Temporomandibular Joints in Juvenile Idiopathic Arthritis. J Oral Maxillofac Surg. 2018. 10.1016/j.joms.2018.06.027 30028954

[18] Kellenberger CJ, Junhasavasdikul T, Tolend M, Doria AS (2018). Temporomandibular joint atlas for detection and grading of juvenile idiopathic arthritis involvement by magnetic resonance imaging. Pediatr Radiol.

[19] Tolend MA, Twilt M, Cron RQ, Tzaribachev N, Guleria S, von Kalle T (2018). Toward establishing a standardized magnetic resonance imaging scoring system for Temporomandibular joints in juvenile idiopathic arthritis. Arthritis Care Res.

[20] Fedorov A, Beichel R, Kalpathy-Cramer J, Finet J, Fillion-Robin J-C, Pujol S (2012). 3D slicer as an image computing platform for the quantitative imaging network. Magn Reson Imaging.

[21] ShapePopulationViewer. RRID:SCR_014167. University of North Carolina at Chapel Hill; North Carolina; USA. http://www.nitrc.org/projects/shapepopviewer/.

[22] Giancane G, Ravelli A (2017). Paediatric rheumatic disease: what is the best definition of clinical remission in JIA?. Nat Rev Rheumatol.

[23] Stoll ML, Cron RQ (2014). Treatment of juvenile idiopathic arthritis: a revolution in care. Pediatr Rheumatol Online J.

[24] Stabrun AE, Larheim TA, Hoyeraal HM (1989). Temporomandibular joint involvement in juvenile rheumatoid arthritis. Clinical diagnostic criteria. Scand J Rheumatol.

[25] Müller L, Kellenberger CJ, Cannizzaro E, Ettlin D, Schraner T, Bolt IB (2009). Early diagnosis of temporomandibular joint involvement in juvenile idiopathic arthritis: a pilot study comparing clinical examination and ultrasound to magnetic resonance imaging. Rheumatology (Oxford).

[26] Koos B, Twilt M, Kyank U, Fischer-Brandies H, Gassling V, Tzaribachev N (2014). Reliability of clinical symptoms in diagnosing Temporomandibular joint arthritis in juvenile idiopathic arthritis. J Rheumatol.

[27] Abramowicz S, Susarla HK, Kim S, Kaban LB (2013). Physical findings associated with active Temporomandibular joint inflammation in children with juvenile idiopathic arthritis. J Oral Maxil Surg.

[28] Billiau AD, Hu YQ, Verdonck A, Carels C, Wouters C (2007). Temporomandibular joint arthritis in juvenile idiopathic arthritis: prevalence, clinical and radiological signs, and relation to dentofacial morphology. J Rheumatol.

[29] Hsieh YJ, Darvann TA, Hermann NV, Larsen P, Liao YF, Kreiborg S. Three-dimensional assessment of facial morphology in children and adolescents with juvenile idiopathic arthritis and moderate to severe TMJ involvement using 3D surface scans. Clin Oral Investig. 2019. 10.1007/s00784-019-02962-5 31168695

[30] Demant S, Hermann NV, Darvann TA, Zak M, Schatz H, Larsen P (2011). 3D analysis of facial asymmetry in subjects with juvenile idiopathic arthritis. Rheumatology (Oxford).

[31] Djordjevic J, Toma AM, Zhurov AI, Richmond S (2014). Three-dimensional quantification of facial symmetry in adolescents using laser surface scanning. Eur J Orthod.

[32] Altman DG, Royston P (2006). The cost of dichotomising continuous variables. BMJ Brit med J.

